# Preemptive iodide treatment in the event of a nuclear disaster: The prepper's guide to the galaxy

**DOI:** 10.1113/EP092154

**Published:** 2025-02-14

**Authors:** Per Karkov Cramon, Søren Holm, Ronan M. G. Berg

**Affiliations:** ^1^ Department of Clinical Physiology and Nuclear Medicine Copenhagen University Hospital ‐ Rigshospitalet Copenhagen Denmark; ^2^ Department of Endocrinology and Metabolism Copenhagen University Hospital ‐ Rigshospitalet Copenhagen Denmark; ^3^ Centre for Physical Activity Research Copenhagen University Hospital ‐ Rigshospitalet Copenhagen Denmark; ^4^ Department of Biomedical Sciences, Faculty of Health and Medical Sciences University of Copenhagen Copenhagen Denmark; ^5^ Neurovascular Research Laboratory, Faculty of Life Sciences and Education University of South Wales Pontypridd UK

Over the past decades, the post‐Cold War optimism that followed the dissolution of the Soviet Union, famously captured by American political scientist Francis Fukuyama, has gradually eroded. Fukuyama's assertion of the ‘ascendancy of Western liberal democracy’ as marking ‘the end of history as such’ no longer reflects the realities of our current era (Fukuyama, 1992). Instead, we find ourselves in a time defined by political and ideological unrest, existential threats to humanity and civilisation, environmental catastrophes, and war. A distinct subculture of so‐called survivalism has thus emerged, referred to as ‘preppers’. These individuals advocate for preparedness in anticipation of worst‐case scenarios – a movement that has proliferated through newsletters, blogs and dedicated stores offering supplies and strategies for survival. Indeed, many European governments now recommend basic preparedness measures for their citizens, such as maintaining various emergency supplies. One pressing concern has been the resurgence of acts of war on the European continent following Russia's invasion of Ukraine in February 2022. Russia, which currently holds approximately 45% of the world's nuclear warheads (Kristensen et al., 2024), has intensified anxieties, also because of targeted bombings near nuclear plants, including Chornobyl. For context, the atomic bombs dropped on Hiroshima and Nagasaki in 1945 had yields of approximately 15 and 21 kilotons of TNT, respectively. In contrast, many modern nuclear weapons are far more powerful, with yields often measured in megatons – thousands of times greater. The devastating consequences of such an event, combined with growing concerns over nuclear plant safety, have led to a renewed focus on protective measures in the event of radiation exposure. One widely discussed recommendation – popular within the prepper community but increasingly supported by some governments and the WHO – is the stockpiling of iodine tablets, often incorrectly referred to as an ‘antidote’ to nuclear radiation. This concern became particularly visible in March 2022, when *The Telegraph* reported a dramatic surge in demand for iodine tablets across the UK and the Oxford Health Company experienced a staggering 15,000% increase in page views for its iodine tablets over the course of a few months (Rees, 2022). By October 2022, *Associated Press* noted that Finnish pharmacies had entirely run out of iodine tablets following recommendations from the country's health ministry that each household purchase a single dose (Associated Press, 2022). Here, we aim to highlight the physical and physiological basis of whether – and how – preemptive iodine administration may offer protection in the event of a nuclear accident, a tale that turns out to be deeply entwined with conflict, weaponry and war.

Most iodine – its name derived from the Greek word ‘iodes’, meaning violet – was forged in the violent birth of the Solar System with the cataclysmic collision of two neutron stars, scattering material into space and creating an environment suitable for the formation of heavy elements (Bartos & Marka, 2019). In such extreme conditions, the so‐called rapid neutron capture process (the r‐process) may occur, in which free neutrons are absorbed into the nucleus of atoms where they are converted into protons (Côté et al., 2021). As more neutrons are captured, heavier nuclei including iodine form – in a stepwise accumulation that forges even the heaviest elements of the periodic table. These newly synthesised elements were deposited into the pre‐solar nebula more than 4.6 billion years ago. Over time, they became part of the molecular cloud that collapsed to form our solar system, including Earth. Looking at the authoritative source of nuclear information, NUDAT3 (NuDat 3, 2025), the nuclear charts show a total of 42 isotopes of iodine (from ^106^I to ^147^I). Forty‐two is a notable number. A curious and poetic coincidence, perhaps, given that the supercomputer Deep Thought famously determined 42 to be ‘the Answer to the Ultimate Question of Life, the Universe, and Everything’ in *The Hitchhiker's Guide to the Galaxy* (1979) by Douglas Adams (1952–2001) (Adams, 1979). Hence, the subtitle of this editorial! Iodine today is present naturally on Earth solely as ^127^I if we neglect the tiny amounts of radioactive ^129^I (*T*_½_ = 16 million years) that is continuously produced in the atmosphere by cosmic radiation, but also present in larger man‐made amounts from bomb testing fallout and nuclear accidents.

The history of iodine is tied to the Napoleonic Wars. By 1811, as the wars were drawing to a close, France faced a severe shortage of gunpowder, and the chemist Bernard Courtois (1777–1838) sought alternative ways to produce potassium nitrate by processing seaweed. During his experiments, Courtois extracted potassium chloride and, after crystallising it, added sulfuric acid to the remaining liquid. To his surprise, the resulting heating produced a striking purple vapour, which condensed into dark, lustrous crystals that he first named ‘substance X’. The word ‘iodine’ was coined 2 years later by the French chemist Joseph Louis Gay Lussac (1778–1850), based on the colour of the vapour. Soon after, a Swiss physician named Jean‐François Coindet (1774–1834), who had previously used burnt sponge and seaweed to treat goitre, took interest in Courtois's discovery, because seaweed had been used for treating goitre as far back as 3600 bc, as recorded in Chinese medical writings, and was also noted in the works of the Greek physician Hippocrates. Coindet suspected that iodine might be the active ingredient responsible for the goitre‐curing properties of seaweed, and subsequently he successfully tested a tincture of iodine on 150 patients, observing a notable reduction in the size of their goitre within 1 week (Coindet, 1820). Already then, the clear link between iodine and thyroid function was thus evident – a link that also renders the gland particularly susceptible in the event of a nuclear disaster.

The thyroid gland is a butterfly‐shaped endocrine organ located anteriorly in the neck, and like iodine, it has war‐related connotations. The English physician and anatomist Thomas Wharton apparently coined the name of the gland in his medical work *Adenographia* of 1656 (Wharton, 1656). The term ‘thyroid’ is likely derived from the Greek word *thyreos*, referring to a large oblong shield often used by Hellenistic armies (Connelly et al., 2022). The principal function of the thyroid is to produce and secrete the thyroid hormones, thyroxine (T_4_) and triiodothyronine (T_3_). These iodine‐containing hormones are essential for development, growth and metabolic homeostasis (Leung et al., 2010). The basic principles of iodide transport in thyroid follicular cells (thyrocytes), including synthesis, storage and secretion, are outlined in Figure 1. Ingested iodine is reduced to iodide and absorbed primarily in the stomach and duodenum. It is then transported through the circulation to the thyroid gland. Varying amounts (5–100%) of the absorbed iodine are taken up by the thyroid, depending on thyroid function and iodine status. Under normal conditions, intrathyroidal iodide concentrations are 20–50 times higher than those in plasma due to the action of the sodium–iodide symporter (NIS), which enables iodide uptake against a steep concentration gradient (Pearce et al., 2004). The remaining iodine is predominantly excreted via the kidneys, with smaller quantities eliminated in stool and sweat (Leung et al., 2010). During nuclear disasters involving fission, such as nuclear power plant accidents or atomic bomb detonations, large quantities of radioactive isotopes are released into the atmosphere. When heavy nuclei like ^235^U or ^239^Pu undergo fission – splitting them into two parts, these resulting nuclei are inevitably highly radioactive (Figure 2, top). A large number of iodine isotopes are created and released in this process (Figure 2, bottom). In nuclear power plant accidents, the volatility of iodine also contributes to the high fractional release. For instance, the Fukushima accident in 2011 resulted in the release of approximately 73 radioactive isotopes (135 when including their radioactive progeny) (Evangeliou et al., 2014). Amongst these are several radioactive isotopes of iodine, notably ^131^I, ^132^I, ^133^I and ^135^I, with half‐lives ranging from approximately 2 h to 8 days. Given that they have identical chemical properties, NIS cannot distinguish them from ^127^I, causing radioactive iodine to accumulate in the thyroid gland alongside stable iodine.

**FIGURE 1:**
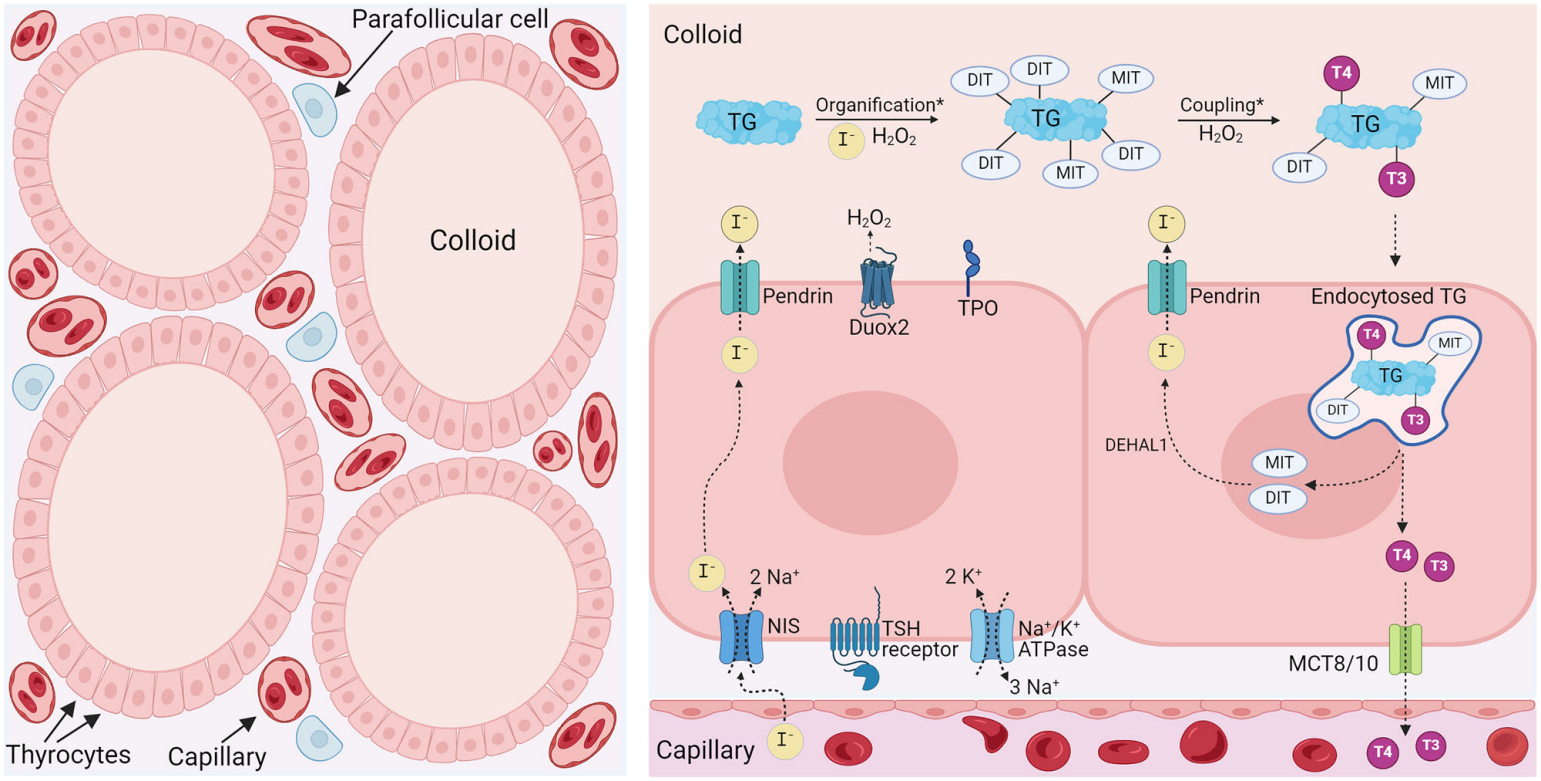
Thyroid histology and physiology. Left: simple illustration of thyroid histology. The thyrocytes are arranged in follicles with colloid in the centre, surrounded by connective tissue, calcitonin secreting parafollicular cells and a dense network of capillaries. The purpose of iodine thyroid blocking is to prevent ^131^I from being taken up by thyrocytes and stored in the colloid. Right: iodine transport in thyrocytes. Thyroid stimulating hormone (TSH) stimulates activity of both Na^+^/K^+^‐ATPase and sodium/iodide symporter (NIS). Iodide is taken up into the thyrocytes via NIS proteins located in the basolateral membrane and transported across the apical membrane via the transmembrane protein pendrin. Inside the follicular lumen, iodide is oxidised and coupled to tyrosine residues on thyroglobulin (TG) to form monoiodotyrosine (MIT) and diiodotyrosine (DIT), and finally, MIT and DIT are proteolysed to form triiodothyronine (T_3_) and thyroxine (T_4_). All the intraluminal steps, marked by *, are catalysed by the enzyme thyroid peroxidase (TPO), located at the apical membrane. When thyroid hormones are required, TSH stimulates thyrocytes to internalise TG by phagocytosis and pinocytosis, TG becomes digested by lysosomes, after which T_3_ and T_4_ are dissociated from TG and eventually released into the blood stream. Iodide bound to MIT and DIT is liberated by a dehalogenase (DEHAL1) and can thereafter be recycled into the follicular lumen and used for hormone synthesis.

**FIGURE 2:**
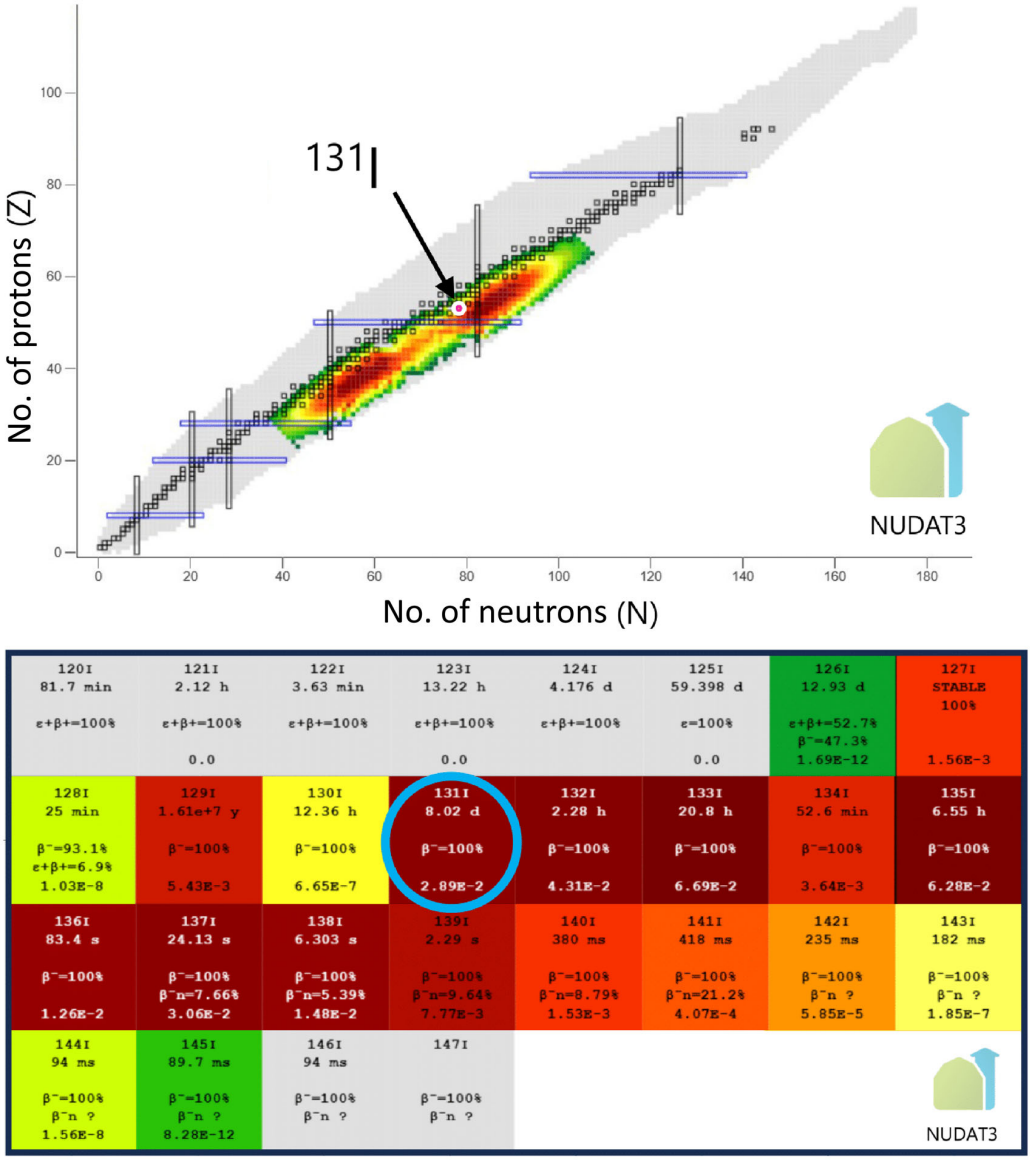
Formation of ^131^I from ^235^U. Top: when a ^235^U nucleus undergoes fission, the result is two nuclei of slightly different size. The initial distribution of the fission products as two ‘clouds’ is here represented in a logarithmic colour scale. The maximum values are on the order of 6% (normalisation to 200%). The location of ^131^I is marked with the arrow. Bottom: the cumulative yield of iodine isotopes from fission of ^235^U. The cumulative yield is higher than the initial yield because it includes decay products from nuclei initially created to the right and below the iodine row. Iodine‐131 is labelled with a blue circle. The numbers shown for each isotope represents (from top to bottom): isotope name, half‐life, mode(s) of decay and (cumulative) yield. Note that the yield represents the number of nuclei, not the activity. To compare activities, values have to be scaled with the decay constant, which is inversely proportional to the half‐life. Ref: https://www.nndc.bnl.gov/nudat3/ [accessed January 9, 2025].

In the following, we focus entirely on ^131^I, as this isotope is the main radioactive iodine isotope posing health concerns following nuclear disasters for several reasons. First, ^131^I is a major fission product of plutonium and uranium, and therefore, large quantities are released into the environment. It is estimated that ∼1760 PBq ^131^I was released in the Chornobyl accident, considered the worst nuclear plant disaster in history. This is enough to treat the entire present human population with 200 MBq radioactive iodine; that is a low standard dose for the treatment of hyperthyroidism (Saenko & Mitsutake, 2024). Second, the half‐life of ^131^I is 8 days; in the event of a nuclear disaster, this is long enough for the isotope to be distributed widely geographically and then ingested and accumulated in the thyroid gland. In contrast, most other iodine fission products have much shorter half‐lives and therefore undergo radioactive decay before they can reach a human body. The thyroid takes up and accumulates ^131^I in the follicular colloid according to the plasma concentration of the radioisotope and thyroid function (see Figure 1). It is well documented that the thyroid is one of the most radiosensitive organs, particularly in children, with the two main risk factors being radiation dose and age of exposure (Iglesias et al., 2017). The increase in thyroid cancer following the Chornobyl accident speaks for itself, as it has been estimated that the number of thyroid cancers attributable to the consequent radiation exposure will exceed 15,000 by the year 2065 (Cardis et al., 2006). The rise in thyroid cancer incidence in relation to the Chornobyl accident has predominantly been observed amongst those exposed as children and adolescents, while there is little evidence of an increased risk for thyroid cancer amongst those exposed as adults (Saenko & Mitsutake, 2024). Accordingly, WHO states that ‘The groups most likely to benefit from iodine thyroid blocking are children, adolescents, pregnant and breastfeeding women, whereas individuals over 40 years of age are less likely to benefit from iodine thyroid blocking’ (WHO, 2017).

Prevention of ^131^I intake following a nuclear disaster requires the routes of exposure to be considered. During a nuclear accident, radioactivity is released into the atmosphere as radioactive plumes, and these are spread geographically according to wind direction and speed. The plumes contain ^131^I in both gaseous and particulate forms, and when inhaled, the blood absorption is high (Crocker, 1984). Inhalation of ^131^I may be the primary exposure pathway for some (Verger et al., 2001), while deposition of radioactive fallout will result in another route of exposure. The latter will cause ^131^I contamination of drinking water, freshwater foods and most importantly plants. Accordingly, the main exposure pathway following the Chornobyl accident was ingestion of fresh milk from cows grazing on contaminated grass. In contrast to a much earlier nuclear accident (Windscale, UK, 1957) where this was acknowledged very early on, no adequate measures were taken in the Chornobyl area. Consequently, children received high thyroid radiation doses, which were also higher than adults because their milk consumption was higher (Saenko & Mitsutake, 2024). Moreover, ^131^I also accumulates in breast milk leading to exposure of newborns. This is likely the reason for the observed rise in thyroid cancer incidence amongst infants after the Chornobyl accident (Unno et al., 2012). Based on the routes of exposure, there are several means to prevent ingestion of ^131^I, such as sheltering (go inside and close windows and doors), evacuation (leave the contaminated areas), and avoidance (of foods that likely contain high amounts of radioisotopes). It may not be possible to avoid inhalation or ingestion of ^131^I, but uptake and accumulation in the thyroid can be minimised with oral administration of stable iodine, so‐called iodine thyroid blocking, usually administered as potassium iodide (KI) tablets (Verger et al., 2001).

For obvious reasons, no randomised clinical trials have been conducted to evaluate the effect of iodine thyroid blocking during nuclear disasters. However, clinical studies have proven that high doses of KI can minimise thyroid uptake of radioactive iodine. The efficacy depends on the KI dose, and it seems that a dose of around 130 mg (∼100 mg iodine) as recommended by WHO is optimal (WHO, 2017). The efficacy of KI administration for thyroid blocking is often reported as the averted dose, defined by the equation: averted thyroid dose = (thyroid dose without blocking − thyroid dose with blocking) / thyroid dose without blocking. When 100 mg or 200 mg KI was administered simultaneously with the radioactive iodine tracer, the dose averted to the thyroid, 24 h after ingestion, exceeded 95% for most study subjects (Blum & Eisenbud, 1967). Increasing the KI dose above this level did not improve the averted ^131^I dose (Koutras & Livadas, 1965). The timing of KI administration is also important. Hence, 100 mg KI has been found to block 98% of thyroid ^131^I uptake when taken simultaneously with the radiotracer, but only 60% was blocked when KI was taken 3 h later, and this blocking effect does decrease steadily. Seventy‐two hours after administration of a single 100 mg KI tablet, only 24% of thyroid ^131^I uptake was blocked (Blum & Eisenbud, 1967). An averted dose above 90% can be maintained by repeated daily administration of 15 mg sodium iodide (∼16.5 mg KI) after initial administration of a single dose of 100 mg sodium iodide (∼111 mg KI) (Sternthal et al., 1980).

So, what is the mechanism of iodine thyroid blocking at the cellular level? There are several possible mechanisms of action. One principal mechanism is often referred to as ‘thyroid saturation’. When plasma iodide suddenly rises to a high concentration, the thyrocytes will take up a high amount resulting in high intrathyroidal iodide concentrations, followed by thyroid uptake blockade (Verger et al., 2001). The blockade is, at least partly, caused by a fast downregulation of the NIS proteins at the basolateral membrane of the thyrocytes (see Figure 1). When rats were acutely exposed to a single intraperitoneal administration of high dose iodide, NIS mRNA was decreased at 6–24 h, whereas NIS protein was decreased only at 24 h (Eng et al., 1999). This downregulation will effectively minimise ^131^I uptake since NIS activity is the major determinant of thyroid iodide uptake rate. Another principal mechanism is isotopic dilution. When stable iodide, that is, ^127^I, is present in plasma in high concentrations, most iodide (including ^131^I) will be excreted via the kidneys rather than taken up in the thyroid. And when the thyroid does take up iodine, it will most likely be in the stable non‐radioactive form. High plasma iodide levels inhibit the organic binding of iodide within the colloid and thus inhibit thyroid hormone synthesis. This phenomenon, known as the Wolff–Chaikoff effect (Eng et al., 1999), may prevent the storage of organified ^131^I in the thyroid colloid and thereby reduce thyroid radiation. Nonetheless, the Wolff–Chaikoff effect could lead to prolonged biological half‐life of ^131^I in the thyroid, and thus increased thyroid radiation, if KI tablets are taken after exposure rather than before. The inhibitory effect is transient. In adults without thyroid disease, thyroid hormone synthesis resumes after 26–50 h (referred to as ‘escape’), even if the iodine overload persists (Wolff & Chaikoff, 1949). Patients with Graves’ disease are more sensitive to the Wolff–Chaikoff effect, and hence, the effect on thyroid hormone synthesis lasts much longer (Burch & Cooper, 2015). Similarly, a recent study demonstrated that high dose stable iodine (∼80 mg KI) given daily for 10 days improved thyroid function in patients with toxic nodular goitre (Hedberg et al., 2024). However, it is a concern that escape from the Wolff–Chaikoff effect can result in severe hyperthyroidism in patients with pre‐existing Graves’ disease or nodular goitre. Lastly, high dose iodine administered daily for 3–14 days to patients with Graves’ disease decreases thyroid vascularity and blood flow (Tsai et al., 2019). This may represent a complementary protective role, since less ^131^I will reach the thyrocytes. It remains to be determined whether iodine thyroid blocking decreases thyroid vascularity and blood flow in individuals without thyroid disease.

Based on the abovementioned kinetic and clinical studies, WHO recommends a single tablet of 130 mg KI in the event of a nuclear disaster. The tablet should be ingested less than 24 h before exposure and not later than 8 h after exposure and is mostly effective in individuals younger than 40 years of age. In case of prolonged exposure, repeated administration of stable iodine may be necessary (WHO, 2017). Some people mistakenly believe that stable iodine is a universal radiation antidote. It is important to stress that stable iodine only protects exposed individuals from radiation‐induced thyroid cancer. It has no effect on other health‐hazardous radioisotopes (such as ^90^Sr, ^134^Cs and ^137^Cs) produced in nuclear disasters.

So, the take‐home message is this: preemptive iodine is not a universal antidote for radiation exposure. It provides protection only against radiation from radioactive iodine isotopes, which are only a subset of the isotopes hazardous to health that are released during a nuclear event. The primary risk to the general population arises from ingesting iodine‐containing foods contaminated with radioactive isotopes, leading to an increased long‐term risk of thyroid cancer. Preemptive iodine should be ingested as a single dose immediately before or at the time of exposure and is particularly relevant for individuals under 40 years of age. It is not for us to determine the relevance of preppers’ strategies to keep iodine tablets stored at home, as long as governmental instructions are followed. Yet, when reflecting on the violent history of iodine – from its cosmic creation to its discovery and now to its therapeutic use for nuclear preparedness – perhaps the message to us all is clear, to resonate the words of John Lennon (1940–1980) and Yoko Ono (b. 1933) when the Cold War was at its height in 1969: Give peace a chance!

## AUTHOR CONTRIBUTIONS

Per Karkov Cramon: conception, first draft, revisions. Søren Holm: first draft, revisions. Ronan M. G. Berg: conception, first draft, revisions. All authors have read and approved the final version of this manuscript and agree to be accountable for all aspects of the work in ensuring that questions related to the accuracy or integrity of any part of the work are appropriately investigated and resolved. All persons designated as authors qualify for authorship, and all those who qualify for authorship are listed.

## CONFLICT OF INTEREST

The authors have no conflict of interest to declare.

## FUNDING INFORMATION

The Centre for Physical Activity Research (CFAS) is supported by TrygFonden (grants ID 101390, ID 20045, ID 125132, and ID 177225). The funders had no role in study design, data collection and analysis, decision to publish, or preparation of the manuscript.
